# The Distinct Effects of Palmitic and Oleic Acid on Pancreatic Beta Cell Function: The Elucidation of Associated Mechanisms and Effector Molecules

**DOI:** 10.3389/fphar.2018.01554

**Published:** 2019-01-21

**Authors:** Miruna Nemecz, Alina Constantin, Madalina Dumitrescu, Nicoleta Alexandru, Alexandru Filippi, Gabriela Tanko, Adriana Georgescu

**Affiliations:** Department of Pathophysiology and Pharmacology, Institute of Cellular Biology and Pathology ‘Nicolae Simionescu’ of Romanian Academy, Bucharest, Romania

**Keywords:** palmitic acid, oleic acid, human pancreatic beta cells, endoplasmic reticulum stress, inflammation, oxidative stress

## Abstract

In this study, we aimed to identify the mechanisms underlying the different effects of palmitic acid and oleic acid on human pancreatic beta cell function. To address this problem, the oxidative stress, endoplasmic reticulum stress, inflammation, apoptosis and their mediator molecules have been investigated in the insulin releasing beta cells exposed to palmitic and/or oleic acid. Herein, we have demonstrated that in cultured 1.1B4 beta cells oleic acid promotes neutral lipid accumulation and insulin secretion, whereas palmitic acid is poorly incorporated into triglyceride and it does not stimulate insulin secretion from human pancreatic islets at physiologically glucose concentrations. In addition, palmitic acid caused: **(1)** oxidative stress through a mechanism involving increases in ROS production and MMP-2 protein expression/gelatinolytic activity associated with down-regulation of SOD2 protein; **(2)** endoplasmic reticulum stress by up-regulation of chaperone BiP protein and unfolded protein response (UPR) transcription factors (eIF2α, ATF6, XBP1u proteins) and by PTP-1B down-regulation in both mRNA and protein levels; **(3)** inflammation through enhanced synthesis of proinflammatory cytokines (IL6, IL8 proteins); and **(4)** apoptosis by enforced proteic expression of CHOP multifunctional transcription factor. Oleic acid alone had opposite effects due to its different capacity of controlling these metabolic pathways, in particular by reduction of the ROS levels and MMP-2 activity, down-regulation of BiP, eIF2α, ATF6, XBP1u, CHOP, IL6, IL8 and by SOD2 and PTP-1B overexpression. The supplementation of saturated palmitic acid with the monounsaturated oleic acid reversed the negative effects of palmitic acid alone regulating insulin secretion from pancreatic beta cells through ROS, MMP-2, ATF6, XBP1u, IL8 reduction and SOD2, PTP-1B activation. Our findings have shown the protective action of oleic acid against palmitic acid on beta cell lipotoxicity through promotion of triglyceride accumulation and insulin secretion and regulation of some effector molecules involved in oxidative stress, endoplasmic reticulum stress, inflammation and apoptosis.

## Introduction

Free fatty acids (FFAs) are essential sources of energy within the cells. They undergo β-oxidation serving to ATP synthesis into mitochondria. Among the three main abundant FFAs, saturated non-esterified fatty acid (palmitic acid, PA) and monounsaturated fatty acid (oleic acid, OA) are the most common. FFAs exert both positive and negative effects on pancreatic beta (β) cell survival and insulin secretory function, depending on concentration, duration, and glucose abundance (Sharma and Alonso, 2014). Changes in physiological plasma levels of FFAs are important for regulation of the β cell function (Haber et al., 2006). Thus, it has been demonstrated that diminution of the plasma FFA levels in either fasted rats or humans, severely impairs glucose-induced insulin release, but PA can augment insulin release in the presence of non-stimulatory concentrations of glucose (Haber et al., 2006). Although, the role of FFA metabolism for insulin secretion stimulation is well known, the regulatory molecular mechanisms controlling the interplay between glucose and fatty acid metabolism and thus insulin secretion are not fully understood. On the other hand, the FFA excess in β cells generates their dysfunction and death affecting the pancreas by a lipotoxic effect. Intracellular mechanisms of lipotoxicity include the accumulation of diacylglycerol (DAG), increased production of ceramide, reactive oxygen species (ROS) generation, endoplasmic reticulum (ER) stress, inflammation, disturbance of intracellular calcium homeostasis, mitochondrial dysfunction and finally cell death (Maedler et al., 2001; Listenberger et al., 2003; Beeharry et al., 2004; Welters et al., 2004).

Significant amount of ROS is constantly generated in β cells in order to accomplish oxygen dependent biosynthetic and secretory function responsible for β cell functionality and regeneration (Trachootham et al., 2008; Jezek et al., 2012; Wang and Wang, 2017). However, the ROS overproduction in pathologic conditions has destructive effects, leading to organellar stress and even to cell death (Liu et al., 2015; Wehinger et al., 2015). Among the four common conditions (hyperglycemia, hyperlipidemia, hypoxia, and ER stress) known to generate ROS in β cells, studies have recently been focused on a more deeply understanding of the oxidative stress-induced lipotoxicity mechanisms (Gerber and Rutter, 2017; Wang and Wang, 2017) mitochondrial fatty acid oxidation and hydrogen peroxide (H_2_O_2_) formation being suggested as key players in ROS generation (Boudina et al., 2007; Lambertucci et al., 2008; Nakamura et al., 2009; Gehrmann et al., 2010; Elsner et al., 2011). Interestingly, matrix metalloproteinase (MMP) 2 may play a crucial role in pancreatic β cell injury induced by excessive oxidative stress (Liu S. et al., 2014).

In many studies, PA has been commonly used to induce both oxidative and ER stress in murine and human β-cells and islets (Cunha et al., 2008; Cnop et al., 2010; Igoillo-Esteve et al., 2010; Hasnain et al., 2014). On the other hand, Ly et al. (2017) have demonstrated that the ROS generation can be corrected by unsaturated fatty acids (such as OA) with a protective effect on ER stress and cytotoxicity. As part of β cell capacity to easily adapt to environment changes, the ER compensatory mechanism activates the cytoprotective response machinery known as unfolded protein response (UPR). This leads in the end to either cell survival or death, by activating three resident molecules within ER: protein kinase R-like ER kinase (PERK), inositol-required enzyme 1 (IRE1) and activating transcriptional factor 6 (ATF6). Physiologically, these three sensors are maintained inactive by association with the chaperone immunoglobulin heavy chain-binding protein (BiP, GRP78). Unfolded protein accumulation within ER generates the sensor release by BiP and activation of the downstream signaling effectors. Alongside GRP78, another resident protein localized on the cytoplasmatic side of ER compartment and also implicated in ER physiology and pathology, is protein – tyrosine phosphatase 1B (PTP-1B). PTP-1B is thought to be an important regulator of glucose homeostasis and body mass.

In different cell types, such as β-cells, the oxidative stress and ER stress activate inflammatory signals (Igoillo-Esteve et al., 2010; Hasnain et al., 2016). Thus, ER stress and inflammation seem to interconnect at multiple levels suggesting that ER stress can be either a potential trigger or a result of chronic inflammation. The ER stress contributes to nuclear factor-kappa beta (NF-kB) activation via both IRE1 and PERK branches (Harding et al., 1999; Jiang et al., 2003; Kaneko et al., 2003; Deng et al., 2004; Han et al., 2009; Nakamura et al., 2011), but it may also induce inflammation by NF-kB-independent mechanisms (Majors et al., 2003; Lauer et al., 2008; Wheeler et al., 2008; Goodall et al., 2010; Mahadevan et al., 2011; Peters and Raghavan, 2011). More than that, the inflammation can alters protein folding with direct consequence on UPR within ER stress (Hasnain et al., 2012). Studies have shown that the inflammatory chemokines and cytokines presented in human pancreatic islets might be generated by PA-induced ER stress (Cunha et al., 2008; Eizirik et al., 2008).

Since saturated fatty acids, especially non-esterified long-chain one (>C14), have been considered to be more toxic than monounsaturated fatty acids, several controversial results regarding dual/contradictory processes responsible for FFAs – dependent β cell function have emerged.

It is therefore justified the necessity to investigate the divergent effects of FFAs in pancreatic insulin secreting cells, in order to fully understand the mechanisms responsible for β cell survival. As a result, the aim of this study was to investigate the effects of palmitic and/or OA on oxidative stress, ER stress, inflammation, apoptosis and on their mediators in human pancreatic β cells.

## Materials and Methods

### Chemicals and Antibodies

The 3-(4,5-dimethylthiazol-2-yl)-2,5-diphenyltetrazolium bromide (MTT) and 2′,7′-dichlorofluorescin diacetate (DCFDA) were purchased from Sigma-Aldrich. The following primary antibodies used were: rabbit polyclonal anti-β-actin, goat anti-PTPase, 1B, rabbit anti-BIP, rabbit anti-GADD 153, rabbit anti-XBP-1, rabbit anti-ATF-6α, rabbit anti-eIF2α, rabbit anti-SOD2, rabbit anti-MMP-2, rabbit anti-TIMP-2, rabbit anti-IL6 and rabbit anti-IL8 (all from Santa Cruz Biotechnology). The secondary antibodies were horseradish peroxidase (HRP)-conjugated anti-rabbit, HRP-conjugated anti-mouse, HRP-conjugated anti-goat (Santa Cruz Biotechnology) and anti-mouse IgG, FITC conjugate from Invitrogen. Laemmli Sample Buffer 2x (SX2) was acquired from Serva and enhanced chemiluminescence reagent Supersignal Ultra from Pierce Biotechnology.

### Cell Culture and FFA Treatment

The human insulin-releasing β cell line designated 1.1B4 was obtained from the European Collection of Authenticated Cell Culture (ECACC, Salisbury, United Kingdom). This was created by electrofusion of immortal human PANC-1 epithelial cell line and freshly isolated human pancreatic beta cells. Cells were grown in 5% CO_2_, 95% O_2_ atmosphere, at 37°C in RPMI-1640 medium containing 11 mM D-glucose supplemented with 10% (v/v) fetal bovine serum (FBS), 2 mM L-glutamine and antibiotics (100 U/ml penicillin, 100 mg/ml streptomycin). All experiments have been performed with cells of passages 8 to 10 in this study.

The preconfluent human pancreatic β-cells were left either untreated (control cells) or treated with FFAs: PA and/or OA, both at a concentration of 250 μM for 24 h (Ahn et al., 2013).

### Cell Viability and Proliferation Assay

Cell proliferation assay was performed by the indirect measurement of cell metabolic activity using MTT (3-(4,5-dimethylthiazol-2-yl)-2,5-diphenyltetrazolium bromide), which detects in the same time the cell viability. Consequently, the 1.1B4 cell proliferation was examined by MTT assay. Briefly, the cells grown in RPMI-1640 medium in either the presence or absence of free FFAs for 24 h, were seeded into 96-well culture plates at a density of 5 × 10^3^ cells per well. After 24 h of culture in serum deprived medium, the MTT solution (0.5 mg/ml) was added to each well for another 4 h. Finally, the supernatant was removed, 100 μl MTT solvent was added to each well and the culture plate was shaken for 10 min before the OD values to be recorded at 570 and 690 nm (as background) at spectrophotometer (TECAN Infinite M200 PRO, Switzerland). All tests have been done in triplicate.

### Nile Red Staining

The lipid droplet staining was carried out following the experimental protocol elaborated by Listenberger and Brown (2007). The 1.1B4 cells were grown in either the presence or absence of 250 μM PA and/or OA, on coverslips for 24 h and fixed in 4% paraformaldehyde in phosphate-buffered saline (PBS) for 15 min, at room temperature (RT). The fixed cells were washed with PBS, and then stained with Nile red (1 μg/ml, Sigma-Aldrich, St Louis, MO, United States) in the dark for 5 min, at RT. The dye in excess was removed by rinsing with PBS and later 2 ng/μl DAPI were used to stain nuclei. The coverslips with the stained cells were mounted up-side down on glass slides and examined by fluorescence microscopy (Zeiss Axiovert epifluorescence microscope).

### Immunofluorescent Staining

This procedure was performed according to previously described method by Popov et al. (2009). The 1.1B4 cells either exposed or not to 250 μM PA and/or OA, were grown until 60–70% confluency on glass coverslips. After washing with PBS for 5 min, three times, the cells were fixed in 2% paraformaldehyde for 20 min at RT, washed again three times with PBS and permeabilized with 0.1% Triton X-100 for 10 min. Then, the cells were incubated with 1% BSA in PBS to block unspecific binding of antibodies. After 30 min at RT, the primary antibody against insulin (1:50 in 1% BSA in PBS) was added overnight at 4°C with rotation. Subsequently, the cells were washed three times with PBS at RT and incubated for 60 min with anti-mouse IgG secondary antibody, FITC conjugate (1:100 in 1% BSA in PBS). The cells were rinsed and mounted on glass slides in one drop of medium containing DAPI (Vectashield, Vector Lab.). The immunolabeled cells were examined by fluorescence microscopy (Zeiss, Observer D1, Germany) and confocal microscopy (Leica Microsystems, TCS SP5, Mannheim, Germany).

### Insulin Quantification Using Enzyme-Linked Immunosorbent Assay (ELISA)

The ELISA method was used to detect and quantify the insulin secreted in the culture media from human pancreatic β cells either stimulated or not with 250 μM PA and/or 250 μM OA for 24 h in 11 mM glucose.

In short, the β cells were grown in RPMI-1640 medium either alone or containing free FFAs (PA and/or OA) for 24 h at 37°C in 5% CO_2_, 95% O_2_ atmosphere. Subsequently, the medium was replaced with serum-free culture medium and the cells were maintained under the same conditions of culture. After 24 h, all the collected media were centrifuged at 150 g for 5 min. The resulting supernatants were preserved at -70°C for insulin quantification. The insulin secretion in culture medium was assayed using a Quantikine^®^ Human, Canine, and Porcine Insulin Immunoassay, a 4.5 h solid-phase ELISA designed to measure insulin in cell culture supernates according to the procedure recommended by the supplier (R&D Systems, Inc.). The assessments were performed in triplicate.

### Detection of Intracellular ROS

The ROS levels were measured in live 1.1B4 cells by a technique that converts DCFDA which is oxidized to a fluorescence dye 2′,7′ –dichlorofluorescein (DCF) (Manea et al., 2005). The fluorescence generated was directly proportional to the amount of oxidized DCFDA to DCF. For this, the cells were seeded in triplicate into 96-well plates at a density of 8 × 10^3^ cells/200 μl. After 24 h of serum starvation, the preconfluent cells were either treated or not with FFAs for other 24 h. Subsequently, the cells were incubated with 10 μM DCF-DA at 37°C in the dark for 1 h, and 0.2 μg/ml Hoechst were added in the last 10 min incubation to stain the nuclei. Next, the cells were washed with Hepes-buffered saline solution (HBSS) containing: 135 mM NaCl, 5.4 mM KCl, 1.8 mM CaCl_2_, 1 mM MgCl_2_, 10 mM glucose, 10 μM Hepes (pH 7.5 at 37°C). The accumulation of DCF (the final oxidation product) in 1.1B4 cells was measured by an increase in fluorescence at 530 nm when the samples were excited at 485 nm using Tecan Infinite M200 PRO Multi-Detection Microplate Reader. The redox state of the samples was monitored by detecting the increase in fluorescence. The ROS production was calculated from the ratio of relative fluorescence units to total DNA levels and expressed as percent.

As for DNA levels, the Hoechst 33342 (2′-[4-ethoxyphenyl]-5-[4-methyl-1-piperazinyl]-2,5′-bi-1H-benzimidazole trihydrochloride trihydrate) was used for the specific staining of the nuclei detected spectrophotometrically in 1.1B4 cells. The Hoechst 33342 is a cell-permeable DNA stain that is excited by ultraviolet light and emits blue fluorescence at 460–490 nm. Hoechst 33342 binds preferentially to adenine-thymine (A-T) regions of DNA. This stain binds into the minor groove of DNA and exhibits distinct fluorescence emission spectra that are dependent on dye: base pair ratios.

### Real-Time Polymerase Chain Reaction (RT-PCR)

Total RNA was isolated from 1.1B4 cells (either treated or not with PA and/or OA) with TRI Reagent (Zymo Research, Irvine, CA, United States) and quantified using a NanoDrop 2000c Spectrophotometer. The cDNA was synthesized from total RNA with High Capacity cDNA Reverse Transcription kit (Applied Biosystems, Foster City, CA, United States). The cDNA was subjected to RT-PCR with species-specific primers for the identification of effector molecules. The RT-PCR was performed using SYBR Green PCR Master Mix (Applied Biosystems) on a ViiA7 RT- PCR system (Applied Biosystems) and Thermal Cycler. Cycling conditions were as follows: an initial denaturation step of 3 min at 95°C followed by 40 cycles of: 3 s at 95°C and 20 s at 60°C. The levels of target mRNAs were normalized to the mRNA level of β-actin as an internal standard. The relative expression of each target gene was calculated using the ddCt method (Livak and Schmittgen, 2001). The PCR products were run in a 2.0% agarose gel and stained with ethidium bromide for visualization and the density of the gel band was determined using the Scion Image for Windows (Scion Corporation, Walkersville, MD, United States).

### Western Blot Analysis

The confluent cells initially seeded at 5 × 10^5^ cells/60 mm dish, were exposed to PA and/or OA for 24 h. The cells were then washed with Tris-buffered saline (TBS) solution containing: 50 mM Tris H-Cl and 150 mM NaCl, pH 7.4 and afterward they were solubilized in 2x Laemmli sample buffer (SX2, Serva, Heidelberg, Germany) containing 4% 2-mercaptoethanol. After protein extraction and clarification of cell lysates by centrifugation, the protein concentration was estimated with an Amido black dye binding assay.

The extracted proteins were resolved by SDS-PAGE by loading 40 μg protein per lane using a 6%, 10% or 12% (w/v) separating gel with a 4% (w/v) stacking gel. After electrophoresis, the proteins were either stained with Coomassie Brilliant or transferred to nitrocellulose membranes (Bio-Rad Laboratories, Hercules, CA, United States). The obtained blots were blocked with 5% (wt/vol) non-fat dry milk or 3% bovine serum albumin (BSA) in TBS containing 0.05% Tween-20 for 1 h.

For detection, the specific antibodies were used at 4°C, overnight at a dilution of 1:200 (for first antibodies) and for 1 h at a dilution of 1:10,000 (for secondary antibodies). Immunoblots were then visualized using enhanced chemiluminescence reagents. Analysis of results was carried out by densitometry with TotalLab 120, non-linear dynamics program, and the value for each band was either normalized to the level of β-actin or expressed as ratio of phosphorylated protein per total protein.

### SDS-PAGE Zymography

Conditioned culture media from 1.1B4 cells (either treated or not with 250 μM PA and/or OA) were analyzed by zymography in order to detect both activated and zymogene forms of MMP-2 and MMP-9 gelatinases and measure their proteolytic activity. Under non-reducing conditions, proteins presented in the conditioned media were subjected to SDS-PAGE electrophoresis. The 10% polyacrylamide gels containing 1 mg/ml gelatine as substrate were used. Following electrophoresis, the proteinase activity was renatured by washing the gel twice for 30 min with 2.5% Triton X-100 to remove SDS. The gels were then incubated for 18 h at 37°C in the buffer containing 50 mM Tris-HCl, 10 mM CaCl_2_, 0.2 mM PMSF, pH 7.4. In order to finally reveal the gelatinolytic activity of MMPs (marked by clear zones against the blue background), 0.2% Coomassie brilliant blue R-250 was used for gel staining and TotalLab TL120 software was utilized to determine relative enzyme activity.

### Data Analysis

All assays were performed in five independent experiments done in triplicate. Data were expressed as mean ± SEM. One-way ANOVA method and GraphPad Prism Software (5.0 version) were used to analyze and compare the data for different experimental conditions. Statistically significant differences were recorded when *P* ≤ 0.05 or *P* ≤ 0.01. The statistical significance, noticeably different, was represented as ^∗^*P* ≤ 0.05, ^∗∗^*P* ≤ 0.01 for values PA/OA/PA + OA effects vs. control, and ^#^*P* ≤ 0.05, ^##^*P* ≤ 0.01 for values OA/PA + OA effects vs. PA effects. The preconfluent human β cells left untreated with FFAs (PA and/or OA) were taken as control.

## Results

### Pancreatic Beta Cell Functionality; Highlighting the Distinct Effects of Palmitic Acid and Oleic Acid

The functional characteristics of β cells were explored *in vitro* either in the absence or in presence of the free FFAs (PA and/or OA) using standardized protocols for proliferation, Nile red staining and insulin secretion.

#### Viability of β Cells After Exposure to Palmitic and Oleic Acid

**FIGURE 1:**
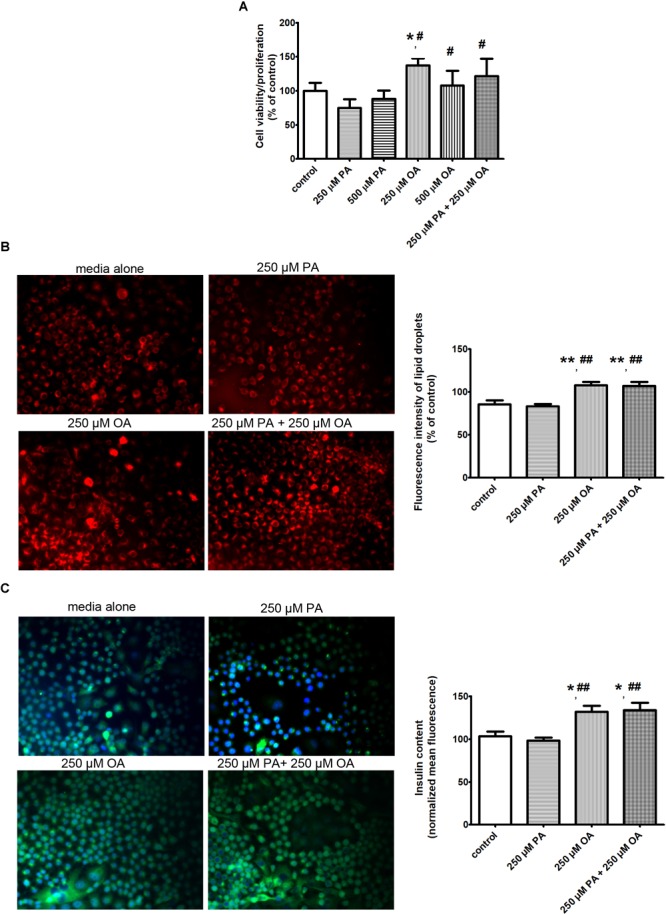
The effects of PA and OA on β cell function in the presence of physiological concentration of 11 mM glucose. **(A)** The β cell proliferation/viability estimated by MTT assay: the cells were incubated in separated experiments with 250 μM PA, 500 μM PA, 250 μM OA, 500 μM OA or 250 μM PA + 250 μM OA for 24 h and dose-dependent effects were recorded. **(B)** The neutral lipid accumulation after FFA supplementation detected by fluorescence microscopy of β cells stained with Nile red: the cells were supplemented with media either alone or containing 250 μM PA, 250 μM OA, or 250 μM PA + 250 μM OA for 24 h. The cells were fixed with paraformaldehyde and stained with Nile red as a marker for neutral lipid. Fluorescence images (20× magnification) using the Nile red fluorescence probe for intracellular lipid content were captured. Higher red fluorescence represents higher lipid content in β cells. **(C)** The insulin secretion from β cells induced by FFAs at physiologically fasting glucose concentrations detected: human islets were incubated at 11 mM glucose either in the absence or in presence of 250 μM PA, 250 μM OA, or 250 μM PA + 250 μM OA for 24 h. Insulin secretion from statically incubated human islets was examined by fluorescence microscopy (20× magnification). Higher green fluorescence represents higher insulin secretion in β cells. Data are shown as mean ± SEM of five independent experiments. The statistical significance, noticeably different, was represented as ^∗^*P* ≤ 0.05, ^∗∗^*P* ≤ 0.01 for values PA/OA/PA + OA effects vs. control, and ^#^*P* ≤ 0.05, ^##^*P* ≤ 0.01 for values OA/PA + OA effects vs. PA effects. The preconfluent human β cells left untreated with FFAs (PA and/or OA) were taken as control.

The cell proliferation/viability was analyzed using MTT assay. For this purpose, the β cells were treated with two different doses of PA (250/500 μM) and/or OA (250/500 μM) for 24 h. As shown in the Figure 1A, in the presence of PA, the cell proliferation/viability was slightly decreased compared to the preconfluent cells left untreated with FFAs and taken as control. In contrast, the OA, the long-chain unsaturated FFA, stimulated the proliferation ability of β cells, suggesting that β cell proliferation was more rapid in the presence of OA (*P* ≤ 0.05, Figure 1A). In addition, the optical density (OD) values were similar for the two doses of OA. The cumulative effect of 250 μM PA and 250 μM OA on β cell proliferation was not stronger than the effect of OA alone, but it was significantly more increased than the effect of PA alone (*P* ≤ 0.05, Figure 1A). With other words, co-treatment with OA improved the effect of PA on β cells proliferation/viability.

#### Oleic Acid but Not Palmitic Acid Increases Neutral Lipid Storage in β Cells

To determine whether the metabolic fate of intracellular 250 μM PA differs in the presence of 250 μM OA, we examined neutral lipid accumulation in β cells after 24 h of FFA supplementation. Neutral lipids were detected by fluorescence microscopy of cells stained with Nile red, a hydrophobic dye that accumulates in lipid droplets. The fluorescent images showed that neutral lipid accumulation was significantly higher after supplementation with either OA alone, or PA and OA together, but not with PA lonely (*P* ≤ 0.01, Figure 1B). Thus, enhanced viability of β cells to either OA, or PA and OA correlates with an increased capacity for accumulation of either neutral lipid or triglycerides.

#### Oleic Acid but Not Palmitic Acid Intensifies Insulin Secretion From β Cells

The FFA- stimulated insulin secretion from human β cells at 11 mM glucose was explored by florescence microscopy and ELISA method (Figure 1C and Table 1). When 250 μM OA were added to culture media, the insulin secretion rate from statically incubated human islets was markedly increased compared to media alone (*P* ≤ 0.05). Just a mild reduction in the insulin secretion was detected in 250 μM PA-treated human islets, but this was notably augmented when 250 μM OA were co-supplemented (*P* ≤ 0.01).

**Table 1:** The analysis of FFA-stimulated insulin secretion in the human pancreatic β cell culture media by ELISA method.

Human β cell cultures	Media alone	250 μM PA	250 μM OA	250 μM PA + 250 μM OA
Insulin (pmol/l)	13.56 ± 0.64	12.87 ± 0.10	16.04 ± 1.01 (^∗^*P* ≤ 0.05) (^#^*P* ≤ 0.05)	15.4 ± 0.18 (^∗^*P* ≤ 0.05) (^#^*P* ≤ 0.05)

*Data are shown as means ± SEM of three independent experiments. The statistical significance, noticeably different, was represented as ^∗^*P* ≤ 0.05, ^∗∗^*P* ≤ 0.01 for values PA/OA/PA + OA effects vs. control, and ^#^*P* ≤ 0.05, ^##^*P* ≤ 0.01 for values OA/PA + OA effects vs. PA effects. The media from preconfluent human β cells left untreated with FFAs (PA and/or OA) were taken as control.*

The results show that OA is capable of enhancing insulin secretion perhaps due to the promotion of neutral lipid storage in β cells, and these effects are associated with their increased viability.

Thus, it is possible that unsaturated FFAs activate signaling pathways which promote triglyceride storage and insulin generation.

### The Metabolic Pathways and Potential Signaling Intermediates Behind the Distinct Effects of Palmitic Acid and Oleic Acid on Pancreatic Beta Cell Function

Because 250 μM PA generated reductions in triglyceride accumulation and insulin secretion in human β cells and these effects were counterbalanced by the addition of 250 μM OA, we sought to investigate the involved metabolic pathways and to determine whether effector molecules differs in the presence of OA co-supplementation. In this sense, the effects of PA and OA on oxidative stress, ER stress, inflammation, apoptosis and on their mediators in human pancreatic 1.1B4 β cells have been explored.

#### Oxidative Stress and Antioxidant Defense

**FIGURE 2:**
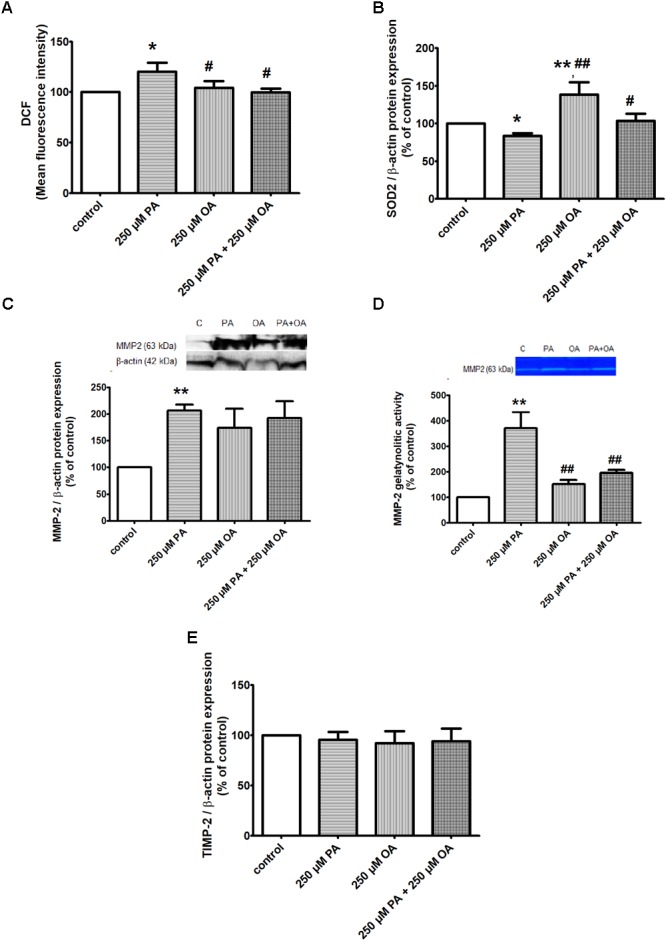
The effects of PA and OA on the redox status of β cell in 11 mM glucose. **(A)** Detection of intracellular ROS production by measuring DCFDA oxidation to DCF in β cells: cells treated for 24 h with PA and/or OA each at a concentration of 250 μM vs. control cells (untreated cells) underwent spectrophotometrically detection of DCF and Hoechst fluorescence within the cells. Intracellular ROS levels were estimated as percent of DCF relative fluorescence units related to total DNA levels. **(B)** Analysis of antioxidant SOD2 protein expression in β cells after 24 h treatment with 250 μM PA, 250 μM OA or their combination. **(C,D)** Evaluation of proteic expression **(C)** and gelatinolytic activity **(D)** of MMP2 in β cells. Preconfluent cells were incubated with PA and/or OA, each at a concentration of 250 μM for 24 h and were subjected to protein expression detection from cell lysate using Western blot technique and investigation of proteolytic activity from conditioned media using SDS-PAGE zymography. **(E)** Proteic expression of TIMP-2 in β cells after 24 h treatment with 250 μM PA and/or OA. Densitometric analysis of SOD2, MMP-2, TIMP-2, was normalized to those of β-actin and the results were expressed as percent of untreated cells. Data are shown as mean ± SEM of five independent experiments. The statistical significance, noticeably different, was represented as ^∗^*P* ≤ 0.05, ^∗∗^*P* ≤ 0.01 for values PA/OA/PA + OA effects vs. control, and ^#^*P* ≤ 0.05, ^##^*P* ≤ 0.01 for values OA/PA + OA effects vs. PA effects. The preconfluent human β cells left untreated with FFAs (PA and/or OA) were taken as control.

The effects of PA and/or OA (250 μM) on intracellular ROS production in β cells were investigated using the fluorescent dye, 2’,7’-dichlorodihydrofluorescein diacetate (H_2_DCF-DA). The results have shown a significant increase in DCF fluorescence when the cells were incubated with 250 μM PA (*P* ≤ 0.05), potentially indicating involvement of ROS in intracellular signaling cascades, while 250 μM OA alone had no effect on the total intracellular ROS production compared to control (Figure 2A). The co-supplementation PA with the monounsaturated FFA oleate significantly diminished DCF fluorescence of the saturated FFA palmitate in pancreatic β cells (*P* ≤ 0.05, Figure 2A). With other words, OA may prevent PA-induced lipotoxicity though inhibition of ROS production and triglyceride storage (showed above).

In correlation with these data, the protein expression of superoxide dismutase 2 (SOD2), a key mitochondrial enzyme involved in quenching ROS, was found to be markedly up-regulated in the presence of 250 μM OA (*P* ≤ 0.01), though PA alone induced a down-regulation of this enzyme (*P* ≤ 0.05) (Figure 2B). But, when the two FFAs were added together on β cells, OA reversed the PA effect on both SOD2 protein expression and total ROS production (*P* ≤ 0.05, Figures 2A,B).

In addition, the activation of MMPs as a result of oxidative stress, and their consequences on different cells and organs have been largely discussed.

In this context, we also investigated the effect of both FFAs on protein expression and gelatinolytic activity of MMP2 in β cells. Compared to control, we observed a significant up-regulation and augmented proteolytic activity of MMP2 after 24 h of incubation with 250 μM PA (*P* ≤ 0.01, Figures 2C,D). Treatment of 1.1B4 cells with 250 μM OA alone led to an un-significant increase in protein expression of MMP-2 and no effect was detected on MMP-2 gelatinolytic activity (Figures 2C,D). When the FFA mixture was used on β cells, it was noticeable the OA capacity to reverse the PA effect on gelatinolytic activity of MMP-2 (*P* ≤ 0.01), and not on its protein expression (Figures 2C,D).

As tissue inhibitor of metalloproteinase-2 (TIMP-2) plays an important role in regulating MMP-2 activity, we decided to investigate FFA effects on TIMP-2 protein expression (Figure 2E). By forming a ternary complex with pro-MMP2 and its activator MMP-14 on the cell surface, TIMP-2 can either initiate or restrain the cleavage and subsequent activation of MMP-2. Our results have shown no differences in TIMP-2 protein expression when the preconfluent human pancreatic β cells were treated with PA and/or OA, both at a concentration of 250 μM for 24 h, compared to untreated (control) cells (Figure 2E).

#### Endoplasmic Reticulum Stress and Apoptosis

**FIGURE 3:**
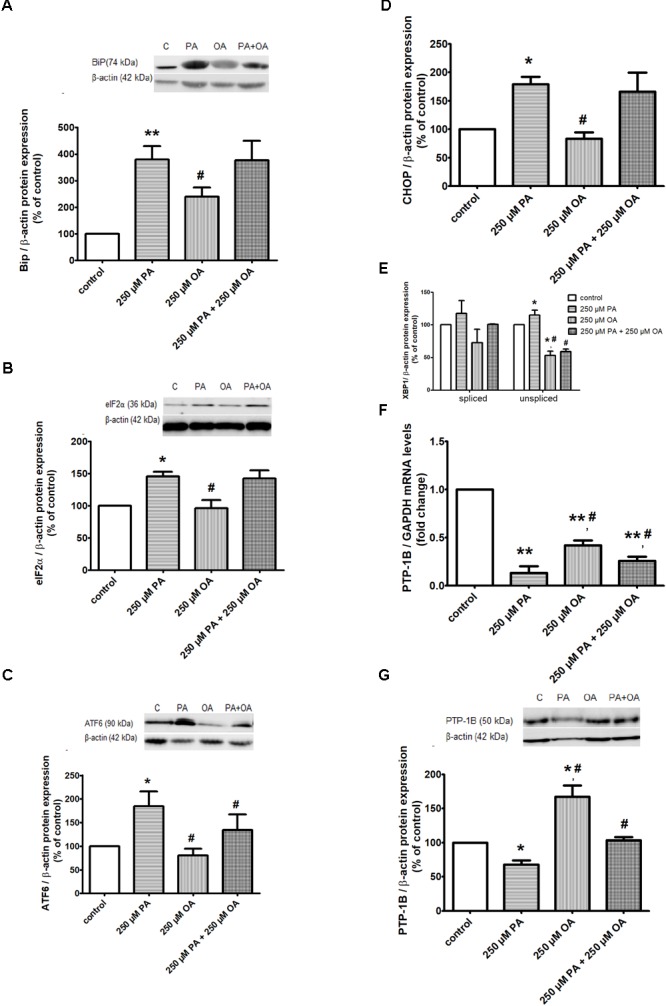
ER proteins in free FFA-treated β cells and control β cells in 11 mM glucose. Proteic expression of Bip chaperone **(A)**, translation initiation factor eIF2α **(B)**, UPR transcriptional factors ATF6 **(C)**, XBP1 **(D)**, CHOP **(E**). The mRNA **(F)** and protein **(G)** expression levels of PTP-1B. The β cells were supplemented with media either alone or containing 250 μM PA, 250 μM OA or 250 μM PA + 250 μM OA for 24 h. All experiments were performed in triplicate. Each protein expression or gene expression was normalized to β-actin, GAPDH, respectively, and plotted as the percentage relative band density. Data are shown as mean ± SEM of five independent experiments. The statistical significance, noticeably different, was represented as ^∗^*P* ≤ 0.05, ^∗∗^*P* ≤ 0.01 for values PA/OA/PA + OA effects vs. control, and ^#^*P* ≤ 0.05, ^##^*P* ≤ 0.01 for values OA/PA + OA effects vs. PA effects. The preconfluent human β cells left untreated with FFAs (PA and/or OA) were taken as control.

To further investigate ER stress response of 1.1B4 cells in the presence of PA and/or OA, the cells were exposed to FFAs for 24 h and several molecular sensors and effectors in UPR were investigated. The obtained data have revealed that 250 μM PA led to a significant up-regulation of BiP chaperone (*P* ≤ 0.01, Figure 3A) and eIF2α, ATF6 and XBP1u isoform UPR transcription factors (*P* ≤ 0.05, Figures 3B–D). Also, it was induced the expression of CHOP multifunctional transcription factor that potentiates the expression of genes responsible for cell death (*P* ≤ 0.05, Figure 3E). For all these ER stress markers, it was observed a significant reversed protein expression when 250 μM OA were added (*P* ≤ 0.05, Figures 3A–D). When XBP1s isoform, another ER stress marker, was investigated, no change in its protein expression was detected after either PA or OA incubation (Figure 3D). Adding the two FFAs together on β cells for 24 h showed that OA inverted the PA effect only for protein expression of ATF6 and XBP1u isoform (Figures 3C,D).

In addition, the ER resident protein, PTP-1B, has shown a significant decrease in both gene (*P* ≤ 0.01) and protein (*P* ≤ 0.05) expression, after treatment with 250 μM PA (Figures 3F,G). On the contrary, the 250 μM OA treatment alone up-regulated the PTP-1B in both mRNA and protein levels (*P* ≤ 0.05, Figures 3F,G). When 250 μM PA were added together with 250 μM OA on β cells for 24 h, it was observed that OA counterbalances the PA effect, up-regulating PTP-1B expression (*P* ≤ 0.05, Figures 3F,G).

#### Inflammatory Processes

**FIGURE 4:**
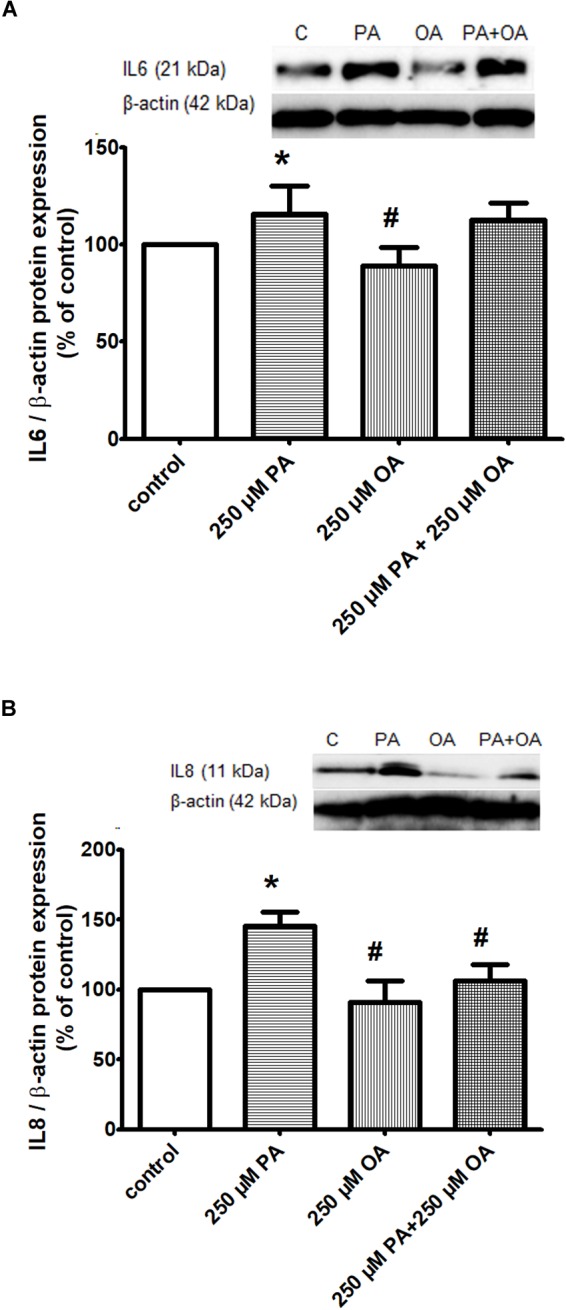
Inflammatory proteins in β cells exposed to FFAs in a physiological concentration of glucose. Proteic expression of inflammatory cytokines IL6 **(A)** and IL8 **(B)** in β cells after 250 μM PA and/or OA treatment for 24 h in 11 mM glucose. The experiments were performed in triplicate. Levels of IL6/IL8 expression were assessed using Western blotting techniques and quantified with ImageJ densiometric analysis. Representative blot (upper panel); quantification of IL6/actin, IL8/actin (lower panel). Data are shown as mean ± SEM of five independent experiments. The statistical significance, noticeably different, was represented as ^∗^*P* ≤ 0.05, ^∗∗^*P* ≤ 0.01 for values PA/OA/PA + OA effects vs. control, and ^#^*P* ≤ 0.05, ^##^*P* ≤ 0.01 for values OA/PA + OA effects vs. PA effects. The preconfluent human β cells left untreated with FFAs (PA and/or OA) were taken as control.

To investigate whether PA can be responsible for inducing an inflammatory process, the 1.1B4 cells were treated with 250 μM PA for 24 h at a pathophysiologically relevant concentration. The results have demonstrated that PA stimulates human β cells to produce significantly increased levels of proinflammatory cytokines IL6 and IL8 (*P* ≤ 0.05, Figures 4A,B). In the case of 250 μM OA treatment, a significantly down-regulation of IL6 and IL8 secretion was induced (*P* ≤ 0.05, Figures 4A,B). The addition of OA to PA resulted in an insignificant reduction of secretion levels of IL6 (Figure 4B), while IL8 levels were significantly decreased (*P* ≤ 0.05, Figure 4B).

In conclusion, the prevention of PA-induced incorporation of saturated phospholipids into the β cell membranes by OA could play a role in the attenuation of ROS production, ER stress, apoptosis, and inflammation.

## Discussion

In this study, we investigated the effects of PA and OA on human pancreatic β cell function and we explored associated mechanisms. Thus, the oxidative stress, ER stress, inflammation, apoptosis and their effector molecules were examined in the insulin releasing 1.1B4 β cells exposed to PA and/or OA. Although various studies analyzed FFA effects on β cells, to our knowledge, this study is the first to demonstrate molecular targets and associated potential pathways.

Our findings show the different effects of saturated PA and monounsaturated OA on pancreatic β cell functionality and on intermediary metabolic and signaling pathways. Specifically, OA had a protective action on β cells reflected by improved parameters of cell function: proliferation, triglyceride accumulation and insulin secretion.

The role of saturated and monounsaturated FFAs at different ambient glucose concentrations on β cell proliferation, apoptosis, and function was investigated by Maedler et al. (2001). Adult rat islets were exposed to palmitic (C16:0) and palmitoleic (16:1) acid alone and in combination, and the results have demonstrated that whereas palmitoleic acid stimulates cell proliferation at normoglycemic glucose concentrations, PA exhibits an inhibitory effect independent of a medium glucose level. In contrast, the monounsaturated palmitoleic acid does not affect cell apoptosis, yet it promotes the cell proliferation at low glucose concentrations, counteracting the negative effects of PA as well as improving cell function. With the induction of apoptosis, the deleterious effects of PA could lead to a reduction in β cell mass, an important determinant of cell functional activity. In agreement with these data, on hepatoma cell lines and on human hepatocyte primary cultures, it has been confirmed that PA is a considerable cytotoxic agent (Moravcova et al., 2015).

As for the accumulation of triglycerides (neutral lipids) induced by OA observed in our study, this could be at least partly explained by the increase in sterol regulatory element-binding protein-1 and peroxisome proliferator-activated receptor gamma expression that act as lipogenic transcription factors (Ricchi et al., 2009). The fact that PA is not able to induce triglyceride synthesis as effectively as OA was documented also in HepG2 cell line (Ricchi et al., 2009) and in rat hepatoma cells H4IIEC3 (Leamy et al., 2014). PA treatment of rat hepatoma cells increased *de novo* lipid biosynthesis resulting in changes in diacylglycerols and phospholipids while OA supplementation ameliorated these changes by increased esterification into triglycerides (Leamy et al., 2014). Another explanation comes from the experiments conducted by Pan et al. (2011) that showed rising concentrations of saturated PA can attenuate the synthesis of triglycerides in goose hepatocytes by an effect on diglyceride acyltransferase from the group of enzymes involved in triglyceride synthesis.

Herein, we demonstrated in cultured β cells that OA promotes neutral lipid accumulation and insulin secretion, whereas PA is poorly incorporated into triglyceride and it does not stimulate insulin secretion from human pancreatic islets at physiologically glucose concentrations. This means that triglyceride accumulation correlates with insulin secretion in β cells exposed to OA. Mixture of OA and PA augmented functional capacity of beta cells reducing their lipotoxicity through promotion of triglyceride accumulation and insulin secretion. Our findings correlate with data that illustrate in a model of cellular lipid metabolism that unsaturated FFAs have a protective function against lipotoxicity though promotion of triglyceride accumulation and insulin generation (Listenberger et al., 2003).

It is generally accepted that FFAs potentiate the insulin secretion at high glucose concentrations (Opara et al., 1994; Gravena et al., 2002). Our study shows that OA and combination of PA with OA enhance the insulin secretion even at low glucose concentrations of 11 mM. Similar to these data, it has been found that short-term exposure of human islets to long-chain FFAs induced insulin secretion at physiologically fasting blood glucose levels, with monounsaturated FFAs (palmitoleate and oleate) being more powerful than saturated FFAs (palmitate and stearate). These effects were partly due to increased glycolytic flux and mitochondrial respiration and partly due to mitochondria independent effects via FFA metabolism and FFAR1/GPR40 signaling (Cen et al., 2016).

In our study, enhanced insulin secretion in the presence of either OA or combination of OA with PA was partly associated with the regulation of effector molecules involved in oxidative stress, ER stress, inflammation and apoptosis. Concerning oxidative stress induced by ROS, it has been shown that it is critically involved in β cell dysfunction during the development of diabetes (Wang and Roper, 2014). Also, the lipotoxicity in β cells occurs through a mechanism involving increases in ROS generation.

Consistent with previous research conducted by other authors, we confirmed that one of the most potent inducer of oxidative stress in pancreatic β cells is PA (Carlsson et al., 1999; Barlow and Affourtit, 2013; Sato et al., 2014), and the molecular mechanisms for cellular ROS production need to be on considered further. In contrast with the PA effect, it has been revealed that OA might both stimulate ROS production and protect from oxidative stress (Ly et al., 2017; Wang and Wang, 2017). Therefore, there are several controverted data debating the influence of monounsaturated FFAs on ROS generation in pancreatic β cells. OA has been described to increase ROS induction by inhibiting mitochondrial respiratory chain (Koshkin et al., 2003), and also by enhancing production of intracellular H_2_O_2_ (Koulajian et al., 2013) in rat smooth muscle cells (Lu et al., 1998) and human hepatoma HepG2 cells (Pang et al., 2015). On the contrary, other studies have revealed that monounsaturated FFAs might prevent formation of H_2_O_2_ by NEFAs in peroxisomes (Gehrmann et al., 2015), as formation of H_2_O_2_ in this compartment, rather than in mitochondria, is believed to be the main mechanism responsible for NEFAs-induced toxicity (Gehrmann et al., 2010; Elsner et al., 2011). Also, OA did not increase mitochondrial ROS level in non-insulin-secreting cells like cardiomyocytes (Joseph et al., 2016) and skeletal muscle cells (Yuzefovych et al., 2010). Moreover, in human coronary artery smooth muscle cells (Lamers et al., 2012) and liver cells (Park et al., 2014) no effect of OA on ROS generation has been observed. Our data demonstrate that exposure of human pancreatic islets to PA caused oxidative stress through a mechanism involving increases in ROS production, MMP-2 protein expression and MMP-2 gelatinolytic activity, and reductions in SOD2 protein expression. On the contrary, OA alone had opposite effects due to its different capacity of controlling these metabolic pathways, in particular act on ROS by stimulating SOD2 expression and reducing MMP-2 activity. No change in TIMP-2 expression in β cells exposed to either PA, OA, or their combination, was recorded. Nevertheless, the supplementation of saturated PA with the monounsaturated OA reversed the effects of PA alone on ROS production, SOD2 expression, MMP-2 activity in pancreatic β cells.

All these data regarding ROS production together with the antioxidant defense exhibited by β cells in the presence of OA, advance important remarks as to whether β cells are/might be entirely capable of fighting against an (oxidative) altered milieu induced by lipotoxicity, using their own environmental lipotoxic elements. Data obtained by former studies on rat pancreatic β cells (Liu C. et al., 2014), attested a certain association between ROS production and MMP-2, results also confirmed by our study. Consequently, we have also observed a possible correlation between oxidative stress induced by PA with the MMP-2 expression and gelatinolytic activity in 1.1B4 cells. Remarkably, OA was able to reverse enhanced MMP-2 proteolytic activity induced by PA. Studies on mouse podocytes (Xu et al., 2015) and L6 skeletal muscle cells (Yuzefovych et al., 2010) reported the ability of OA either to reduce or even to abolish PA-induced ROS formation when the two fatty acids were mixed together.

Besides all of these, we have found that saturated PA induced the ER stress by proteic expression up-regulation of chaperone BiP, UPR transcription factors (eIF2α, ATF6, XBP1u), and by PTP1B down-regulation in both mRNA and protein levels.

Since resident molecules that mediate UPR response and their downstream molecules have been demonstrated to be up-regulated in the presence of FFAs, several mechanisms have been described in order to better understand lipotoxicity induced ER stress. One can assume that both PA and OA affect Ca^2+^ homeostasis in the ER causing protein misfolding and ER stress independent of oxidative stress (Cnop et al., 2010), even though OA induces a much milder ER stress than PA (Cunha et al., 2008; Yuzefovych et al., 2010; Xu et al., 2015; Joseph et al., 2016). Other authors indicate a PA associated rapid increase of saturated lipid content in the ER, simultaneous with an altered ER morphology and integrity resulting in ER stress (Borradaile et al., 2006; Pineau and Ferreira, 2010; Boslem et al., 2011). Recently, it has been attested that a basal expression of ATF6α is essential for β cell survival in physiological conditions. Moreover, ATF6α induces the expression of cluster of genes involved in protein folding, like chaperone BiP (Yamamoto et al., 2007). In addition, the constant production of spliced XBP1 induces β cells dysfunction and apoptosis (Iwawaki et al., 2010), XBP1 splicing being the central event of the activation of IRE1α, one of the three sensors of the UPR. Sometimes function of ATF6α has been demonstrated to act dependent to XBP1, modulating the expression of genes involved in the protein degradation (Yamamoto et al., 2007). In our study, we also observed a significant effect in the proteic expression of eIF2α in the presence of each of FFAs used. This may indicate a mild protein load in the ER after cell exposure to the agents. Several studies have attested an increase in mRNA expression (Vasu et al., 2013) and protein phosphorylation of eIF2α in pancreatic β cells treated with PA (Karaskov et al., 2006; Cunha et al., 2008; Martinez et al., 2008; Bachar et al., 2009; Cunha et al., 2009; Gwiazda et al., 2009).

Our data have shown that both FFAs act like stressors to 1.1B4 cells, generating different responses. We therefore have demonstrated that in the presence of PA alone a significant increase of molecular chaperone BiP and ER stress effectors (eIF2α, ATF6, XBP1u) protein expression was induced. This is an important observation because it is well known that up-regulation of molecular chaperones is characteristic to ER response in many cell types including β cells. Several studies have reported up-regulation of molecular chaperones in rat PA-treated β cells (Kharroubi et al., 2004; Cunha et al., 2008; Martinez et al., 2008; Hellemans et al., 2009).

Importantly, our study further demonstrated that OA alone led to significant decreases in all of these molecule expressions with role in ER stress. It needs to be mentioned that, the addition of OA to PA generated the significant changes to the PA response alone only at the level of ATF6 and XBP1u proteins.

Because in ER stress there are involved not only the unfolded proteins from cytoplasmic compartment (eIF2α, ATF6, XBP1) but also those from nucleus compartment, we analyzed CHOP molecule that is a component of the downstream effectors of ATF6 as well, which potentiates the expression of genes responsible for cell death. There are experimental evidence suggesting that inhibition of ER stress proteins (like CHOP) has beneficial effects on PA induced apoptosis in several cell types (Pfaffenbach et al., 2010; Sieber et al., 2010; Malhi et al., 2013) including β cells (Boyce et al., 2005; Cnop et al., 2010). The UPR is essential for mitigating ER stress and protecting cells from apoptosis. Under ER stress, CHOP positively controls the expression of genes involved in apoptosis (Novoa et al., 2001; Kojima et al., 2003; Marciniak et al., 2004; Yamaguchi and Wang, 2004; Ohoka et al., 2005). In physiological conditions, CHOP is down-regulated and is strongly up-regulated in ER stress, its generation being controlled by ATF4 (Harding et al., 2000; Ma et al., 2002) and ATF6 (Okada et al., 2002; Gu et al., 2004; Adachi et al., 2008; Song et al., 2008). In agreement with these data, we have found that in human pancreatic β cells the CHOP protein expression was up-regulated by PA, down-regulated by OA treatment and unchanged by PA and OA mixture. These results suggest that either PA or OA, via eIF2α, ATF6, XBP1u transcription factors in UPR signaling and CHOP activation, lead to the expression of genes required for amino-acid metabolism, oxidant/antioxidant response and apoptosis. It is important to note that most UPR molecules have an adaptative function in β cells, and the delimitation between survival and apoptosis is determined by the intensity and duration of ER stress stimuli and by the cell response as well.

In addition, prior studies attested that the ER resident protein, PTP-1B, has different substrates and regulates distinct branches of ER stress signaling. As a result, PTP-1B produces individual effects in response to several ER stress inducers in a cell-dependent manner. Therefore, in mouse embryonic fibroblasts, lack of PTP-1B triggers impaired ER stress-induced IRE1 signaling and reduced apoptosis (Gu et al., 2004), while in liver, PTP-1B deficiency reduced the activation of PERK/eIF2α and protein synthesis (Delibegovic et al., 2009; Agouni et al., 2011). On the contrary, other studies have shown that the absence of PTP-1B may cause enhanced PERK/eIF2α phosphorylation in MIN6 insulinoma β cells and brown adipocytes, whereas PTP-1B overexpression inhibits ER stress response (Bettaieb et al., 2012). Additionally, Krishnan et al. (2011) confirmed that the PTP-1B inhibition promotes PERK activity during cellular response to ER stress. The activity of PTP-1B is known to be regulated by inhibitory process like sulfhydration. In turn, PERK activation lead to generation of endogenous hydrogen sulfide pointing the reversibly inactivation of PTP-1B (Krishnan et al., 2011). Islet function may be modulated by PTP-1B deficiency through an ER stress response (Liu S. et al., 2014). Our results have shown a significant decrease in PTP-1B gene and protein expression after incubation with PA, meaning that even a mild ER stress might be dependent of PTP-1B modulation. OA treatment had an opposite effect on PTP-1B expression reducing even PA effect on human pancreatic β cells. There are studies that demonstrate that both saturated and unsaturated FFAs-dependent inhibition of PTP-1B may be correlated with Pi3K/PDK1/Akt activation (Shibata et al., 2013).

Additionally, our present data have shown that the IL6 levels were significantly induced following PA treatment. In this context, we suggested that PA may also contribute to β cell inflammation via increasing IL6 secretion. We also have found that IL8 levels were increased following PA treatment, which are also critically important in β cell inflammation. These two proinflammatory cytokines not only amplify the NF-κB signaling pathways that originally led to their production through cell surface receptor activation (an autocrine loop), but also will stimulate nearby cells in a paracrine manner. The OA exposure also resulted in a significant decrease of IL6 and IL8 levels. Interestingly, PA induced cell proliferative effect was attenuated by the addition of OA in our β cell culture system, but significant reductions were found only for IL8 expression. It could be suggested a potential correlation between raised IL6, IL8 expression and decrement in insulin secretion in the pancreatic β cells treated with PA. Whether the inflammation causes loss of functional β cell mass and thereby contributes to the development and progression of type 2 diabetes remains to be elucidated.

Our results are in agreement with several studies that suggest a link between these three proinflammatory cytokines and PA induced inflammation. Thus, it has been demonstrated that after 24 h and even 48 h, PA led to protein secretion of IL6 and increased gene expression of both IL6 and IL8 in human islets (Igoillo-Esteve et al., 2010). More experimental evidence have shown the absence of OA effect in human islets too (Igoillo-Esteve et al., 2010), while data on intestinal epithelial cells indicated increased IL6 mRNA levels after OA treatment (Yoshida et al., 2001).

**FIGURE 5:**
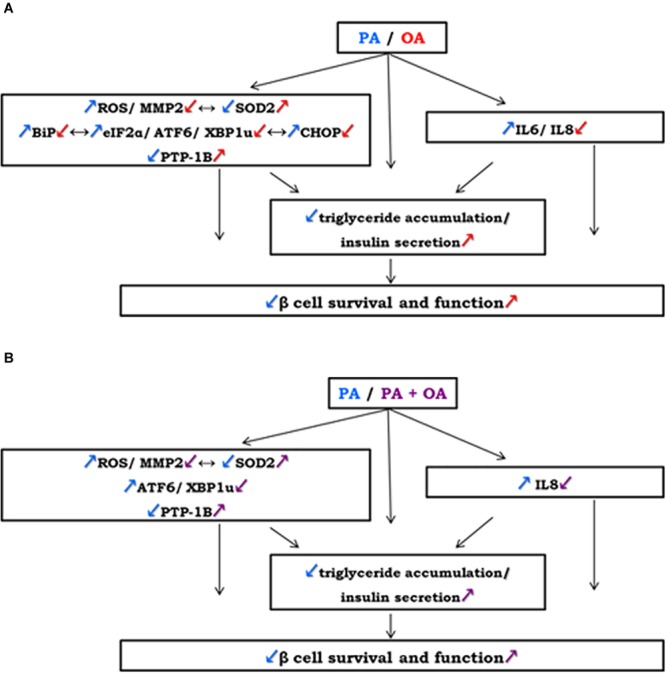
A potential mechanism of OA regulating human β cell survival and function by linking oxidative stress with ER stress, apoptosis and inflammation within the cells: **(A)** on the contrary to 250 μM PA, 250 μM OA exert: an increasement of antioxidant SOD2 correlated with lowered ROS levels and MMP-2 activity; down-regulation of UPR-related transcriptional factors (BiP, eIF2α, ATF6, XBP1u, CHOP) and of inflammatory cytokines (IL6, IL8); up-regulation of ER regulating enzyme PTP-1B. **(B)** Co-supplementation of 250 μM PA with 250 μM OA reversed the effects of PA alone regulating insulin secretion from pancreatic β cells through ROS, MMP-2, ATF6, XBP1u, IL8 reduction and SOD2, PTP-1B activation. These effects may be associated with an increasement in insulin secretion and accumulation of neutral lipids in β cells leading to β cell regulation by OA treatment.

In conclusion, our study clearly demonstrates the distinct effects of PA and OA on pancreatic β cell function and reveals the associated mechanisms and effector molecules. Thus, OA activates signaling pathways that promote triglyceride storage and insulin generation, whereas PA is poorly incorporated into triglyceride and it does not stimulate the insulin secretion. OA has opposite effects due to its different capacity of controlling some metabolic pathways, it attenuates: ROS production, ER stress, apoptosis and inflammation via the SOD2 and PTP1B overexpression, reduction of the ROS levels and MMP-2 activity, and up-regulation of chaperone BiP, UPR transcription factors (eIF2α, ATF6, XBP1u, CHOP) and of proinflammatory cytokines (IL6, IL8). A potential mechanism of OA regulating human β cell survival and function by linking oxidative stress with ER stress, apoptosis and inflammation within the cells was schematized in the Figure 5.

Moreover, the study shows the protective action of OA against PA on β cell lipotoxicity through promotion of triglyceride accumulation and insulin secretion and regulation some effector molecules involved in, particularly by ROS, MMP-2, ATF6, XBP1u, IL8 reduction and SOD2, PTP-1B activation.

In conclusion, the prevention of PA-induced incorporation of saturated phospholipids into the β cell membranes by OA could play a role in the attenuation of ROS production, ER stress, apoptosis and inflammation.

Our findings suggest that treatments with monounsaturated FFAs designed to limit oxidative stress, ER stress, inflammation and apoptosis may point toward novel strategies for improving β cell function under saturated conditions.

## Author Contributions

MN performed the *in vitro* experiments and data acquisition, analysis and interpretation of data, and wrote the manuscript. AC was effectively involved in conducting the experiments. GT participated in designing the study. MD carried out the Western blot and RT-PCR analysis. NA contributed to the acquisition of the fluorescence images. AF performed the statistical analysis. AG made substantial contributions to the conception and design of the study, helped to draft the manuscript and revised it critically regarding important intellectual content.

## Conflict of Interest Statement

The authors declare that the research was conducted in the absence of any commercial or financial relationships that could be construed as a potential conflict of interest.
